# Quantitative analysis of the gaze and the kinetic/kinematic evaluation of expert and novice physical therapists during standing/sitting assistance: a pilot study

**DOI:** 10.3389/fresc.2024.1426699

**Published:** 2024-11-21

**Authors:** Satoru Sekine, Yoshimi Sakurai, Yoshitsugu Omori, Yuji Morio, Junichi Yamamoto

**Affiliations:** ^1^ Medical Informatics, Tottori University Hospital, Yonago, Tottori, Japan; ^2^ Faculty of Systems Design, Tokyo Metropolitan University, Hachioji, Tokyo, Japan; ^3^ Faculty of Medical Sciences, Shonan University of Medical Sciences, Yokohama, Kanagawa, Japan

**Keywords:** expert, novice, physical therapist, eye track, motion capture, standing up

## Abstract

**Introduction:**

In rehabilitation practices, expert therapists are believed to proficiently observe and assist patients. However, limited research has quantified the gaze behaviors of physical therapists during patient support. This study investigated the gaze patterns of expert and novice physical therapists from a first-person perspective during the process of assisting collaborators to stand. The aim was to determine which body parts received prolonged attention and to explore the characteristics of the support provided.

**Methods:**

Seven experienced physical therapists were recruited as expert participants, and 17 physical therapy students served as novice participants. We also recruited additional students as collaborators and asked them to behave as if they were patients. Both expert and novice participants wore a wearable eye tracker while assisting the collaborators to stand. We analyzed the gaze focus on specific body parts and the center of mass sway of the collaborators.

**Results:**

Experts spent 10.75% of the total time gazing at the head area, compared to 4.06% for novices, with experts displaying significantly longer gaze durations (*p* < .05). Furthermore, there was a significant difference in the number of gaze fixations, with experts averaging 25.71 fixations and novices 8.65 (*p* < .05). Experts also facilitated a slower sway in the collaborator's center of mass (0.44 m/s for experts vs. 0.49 m/s for novices; *p* < .01) and positioned the collaborator with a more pronounced trunk flexion during sitting and standing transitions (41.0 degrees for experts vs. 37.8 degrees for novices; *p* < .01).

**Discussion:**

The findings suggest that experts may monitor the collaborator's center of mass position by focusing on the head area. Properly positioning the head forward may allow for optimal forward movement of the center of mass, potentially reducing the effort required by the collaborator to stand. This study is the first to explore differences in support strategies through the measurement of physical therapists’ gaze during assistance.

## 1 Introduction

The ability to independently stand up is crucial for executing various basic activities of daily living, such as toileting and bathing, and serves as a foundational skill for ambulation (1). When performing the sit-to-stand movement, it is necessary to lean the trunk forward and adequately shift the center of mass (COM) between the base of support (BOS), then lift pelvis from the seat while coordinating the extension of both the trunk and lower limbs. Moreover, unassisted standing is pivotal for maintaining independence in daily life and plays an important role in fall prevention (1, 2). However, populations such as the elderly and those with sensory or neurological impairments are at an elevated risk of falls. Contributing factors include decreased muscle strength, balance and gait impairments, and declines in visual or cognitive function, which are particularly common in the elderly (3). Therefore, regaining stable standing ability is one of the primary goals of rehabilitation, and providing assistance with standing is frequently incorporated into daily living and therapeutic exercises. Several studies have demonstrated that supporting the standing process can improve self-care, mobility, and balance skills (4, 5). During these exercises, therapists must not only recognize early indicators of potential falls but also understand the optimal methods to support the body to mitigate fall risks. It is essential to effectively monitor a patient's body tilt and the COM and to coordinate the timing of support accurately to enhance patient safety. In this context, eye tracking technology becomes invaluable, offering an objective and quantitative means to assess the focus and monitoring techniques employed by therapists.

**Figure 1:**
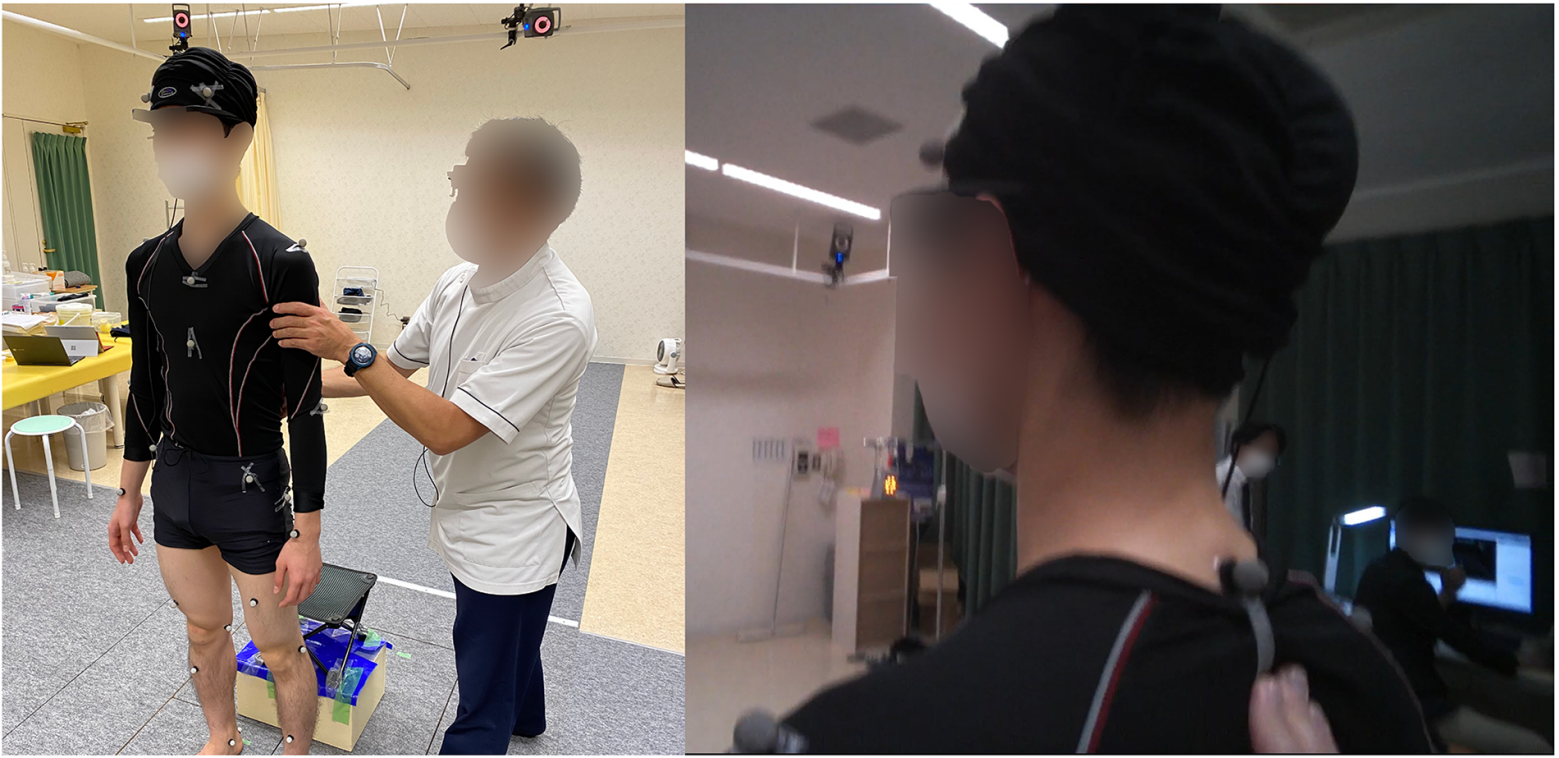
The third-person of view and the first-person of view. The third-person view refers to a photo or video taken from a different location of a scene including the operator. The first-person view refers to a photo or video taken from the viewpoint of the operator.

Eye tracking technology can be categorized into two main types: screen-based and wearable eye trackers, each differing in their visual capabilities and functionalities. Screen-based eye trackers typically employ an infrared sensor and camera mounted on or near a computer screen, capturing the participant's eye movements as they engage with visual stimuli displayed on the monitor. These devices are stationary, necessitating that the observer remains seated in front of the monitor, thereby limiting their mobility and restricting their use primarily to laboratory settings. The imagery captured is often from a third-person perspective, filmed by a videographer positioned externally to the scene, providing a distant overview that allows participants to discern the spatial relationships within the environment (Figure 1; left panel). Conversely, wearable eye trackers incorporate the technology into lightweight glasses or head-mounted devices, merging the roles of participant and videographer into a single entity. This integration permits free movement and interaction with the surroundings, with the eye tracking data continuously recorded. Wearable devices offer enhanced flexibility and mobility, enabling the study of visual attention and behavior in dynamic real-world contexts such as outdoor activities, social interactions, and direct task engagement. The visuals obtained from wearable eye trackers are typically from a first-person perspective (Figure 1; right panel). The wearable eye tracker, worn directly by the observer, offers a direct observational viewpoint, thereby providing an authentic representation of the scene as it would appear to the observer themselves. Research has shown that gaze behaviors markedly differ between viewing scenarios facilitated by screen-based vs. wearable eye trackers (6, 7). This variation is attributed to the natural differences in head movements and focal points encountered in live situations as opposed to static, screen-based viewing. Consequently, using wearable eye trackers enhances the ability to accurately capture therapists’ gaze data in active support scenarios.

In recent years, a substantial body of research within the medical field has utilized eye tracking technology to analyze the gaze patterns of experts and novices, aiming to delineate differences between these groups. Several studies have focused on monitoring these disparities through the gaze of novice users, applying these insights in educational settings. For instance, Gegenfurtner et al. (8) instructed novice medical students on specific focal points used by experienced radiologists when analyzing x-ray images for diagnostics. Similarly, a wearable eye tracker was employed in a clinical anatomy course to demonstrate to students the specific areas an instructor observes during laparoscopic surgery procedures (9). These studies underscore the efficacy of eye tracking technology as an invaluable tool in enhancing medical education.

Within the medical field, screen-based eye tracking has been instrumental in elucidating how experts efficiently resolve complex tasks by analyzing eye movement parameters. Notably, various studies have compared the gaze patterns of experts and novices, demonstrating that experts can assimilate information within the same or a shorter timeframe (10, 11). These investigations have focused on gaze data collected during the viewing of angiographic videos and x-ray images. For example, Giovinco et al. (11) found that experts devoted more time to gazing at areas relevant to the task, while Brunyé et al. (10) observed similar patterns in task-relevant areas. However, it is important to note that the stimuli utilized in these studies were not captured by the observers themselves, and therefore, might not fully represent their clinical expertise.

Numerous studies in the medical field have employed wearable eye trackers to assess eye movements during simulated surgeries, revealing findings distinct from those using screen-based eye trackers. Wilson et al. (12) equipped both expert and novice surgeons with wearable eye trackers to perform simulated laparoscopic surgeries, finding that experts focused on the surgical task for longer durations than novices. Similarly, Tien et al. (13) analyzed the gaze patterns of expert and novice surgeons during simulated procedures and noted that experts checked the patient's vital signs more frequently than novices. These studies (12, 13), which measured gaze during active experimental tasks, present gaze characteristics that diverge from those observed in studies using only screen-based methods, where the patient is merely monitored on a display. When actively assisting a patient, experts were found to focus on task-relevant areas for similar or longer periods compared to novices with screen-based eye trackers; however, with wearable trackers, experts consistently maintained their focus on these critical areas for extended times. The variation in gaze duration attributed to differing viewpoints underscores the potential of wearable eye trackers for conducting research with higher ecological validity.

In the field of rehabilitation, Hayashi et al. (14) explored gaze measurement by employing a wearable eye tracker to capture a third-person view. In their study, novice and expert physical therapists were instructed to observe a video of a patient walking. The findings indicated that experts identified more gait abnormalities and spent less time fixating on the task-relevant areas (e.g., head) than novices. This suggests that experts possess the ability to rapidly discern critical diagnostic features, thereby gathering information more efficiently. However, it is noteworthy that this experiment involved participants wearing wearable eye trackers while viewing a video on a screen, thus mirroring conditions akin to those found in studies using screen-based eye trackers. Given the different findings associated with screen-based and wearable eye trackers in the medical domain, it is plausible that observing patients directly in a real-world setting may yield different results.

In the realm of rehabilitation, it is feasible to analyze differences that approximate clinical practice by measuring eye gaze from first-person perspectives during actual support scenarios involving both experts and novices. Standing and sitting movements are evaluated using indicators such as the forward tilt of the trunk, the velocity of the COM shift, and the spatial relationship between the feet and the COM (15). Proper execution of these movements necessitates pushing the hips forward after the patient's COM has advanced. This approach prevents the need for patients to generate momentum using their entire body to move the COM forward, as standing from a position where the COM is behind requires considerable effort and can be burdensome (16). Therefore, therapists must meticulously monitor the patient's body tilt, COM position, and COM shift velocity, providing support synchronized with the patient's movements. We hypothesized that experts render assistance based on such critical information. However, no study has concurrently measured both patient body movements and expert gaze, indicating an area for further research. In this study, both expert and novice physical therapists were equipped with wearable eye trackers to ascertain their gaze focus during support activities. Additionally, the collaborators’ standing and sitting movements were captured using motion capture and ground reaction force plates, aiming to elucidate how experts assess the position and velocity of the COM.

## 2 Materials and methods

This study was approved by the Research Ethics Committee of Shonan University of Medical Sciences.

### 2.1 Equipment and materials

**Figure 2:**
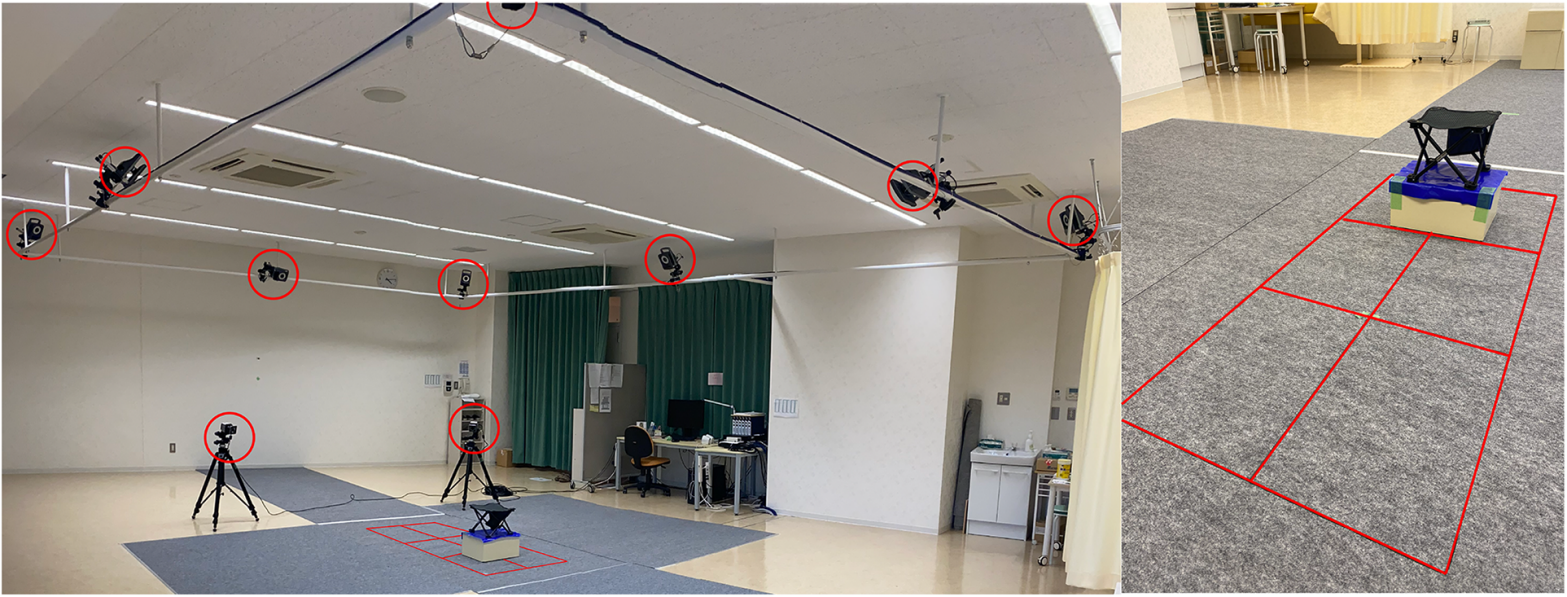
Settings of the experiment. Circles indicate the position of the motion capture camera. Squares indicate the location of the ground reaction force plates. The ground reaction force plates were arranged in a 2 × 3 configuration. Chairs were placed at the edges of the two rear ground reaction force plates.

Figure 2 shows the experimental setup. The experiment was conducted in the analysis laboratory of the university.

A three-dimensional motion analyzer consisting of eight optical reflection cameras (T-20S camera, VICON) and two optical reflection cameras (Vero2.2 camera, VICON) and six ground reaction force plates (AMTI, 400 mm × 600 mm) were used to measure the motion. A chair with a height of 41.5 cm was placed on top of the two rear ground reaction force plates. The sampling frequency of the three-dimensional motion analyzer was 100 Hz and that of the ground reaction force plates were 1,000 Hz. The data obtained from the three-dimensional motion analyzer and the ground reaction force plates were synchronized on the computer. The data sampled from the ground reaction force plates was downsampled from 1,000 Hz to match the timestamp of the motion analyzer data, resulting in a temporal resolution of 10 ms. The timing of the start and end of each experiment was determined by the Motion Analyzer signal, and calibration was performed before each experiment.

To measure the gaze of both expert and novice participants during the support activities, wearable eye trackers (Tobii Pro Glasses 3, Tobii) were utilized. Prior to the experiment, each participant underwent a calibration process using the Tobii Pro Glasses 3 Controller (Tobii). The eye trackers operated at a sampling frequency of 50 Hz, employing near-infrared light to capture reflection patterns from the cornea and pupil, which facilitated the estimation of the gaze point. A single calibration point, as specified by Tobii's calibration method, was used. Calibration was deemed successful if the target card presented and the participant's gaze coincided for the duration specified by the Tobii Pro Glasses 3 Controller settings.

**Figure 3:**
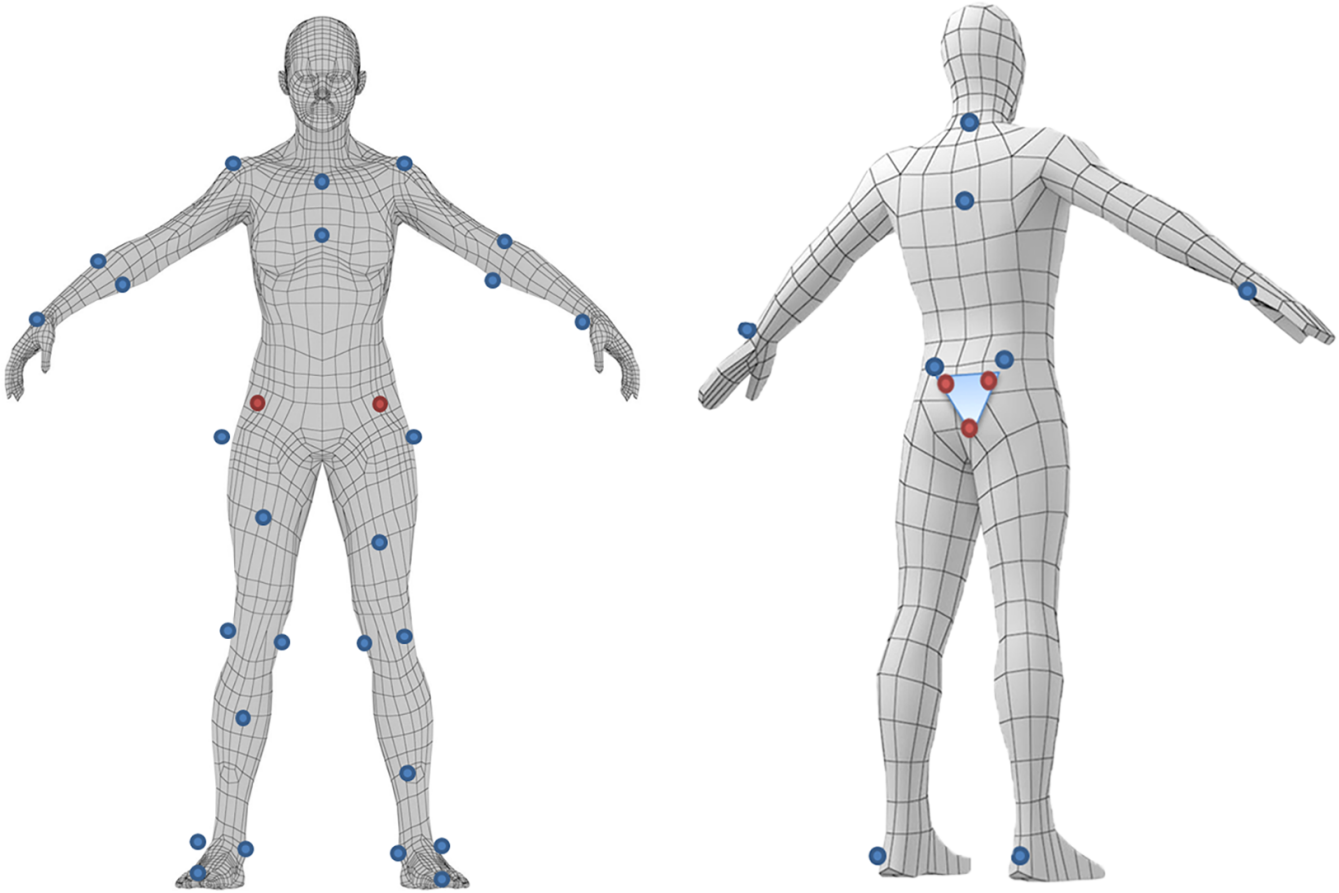
Position where marker is attached. Infrared markers are attached to the body to identify the body motion by motion capture.

Infrared reflective markers were strategically positioned at 34 key locations on the body and three additional points on a jig fabricated according to the VICON Plug-in Gait model (Figure 3). For defining the trunk segment, markers were affixed to the jugular notch, the xiphoid process, the spinous process of the second thoracic vertebra, and the spinous process of the seventh thoracic vertebra. To define the upper extremity position, the bilateral acromion, the bilateral epicondyles of humerus, and the bilateral styloid process of radius and ulna were affixed to the center of the ulnar process. For the lower extremity segments, markers were applied to critical points including both hip joints (specifically, the distal one-third between the superior anterior iliac spine and the greater trochanter), both thighs, both knee joints (medially and laterally positioned at the center of the patella, excluding the patella, at the level of the patellar midpoint), both lower legs, both lateral and medial malleolus, both ankles, both head of second metatarsal, and both head of fifth metatarsal.

**Figure 4:**
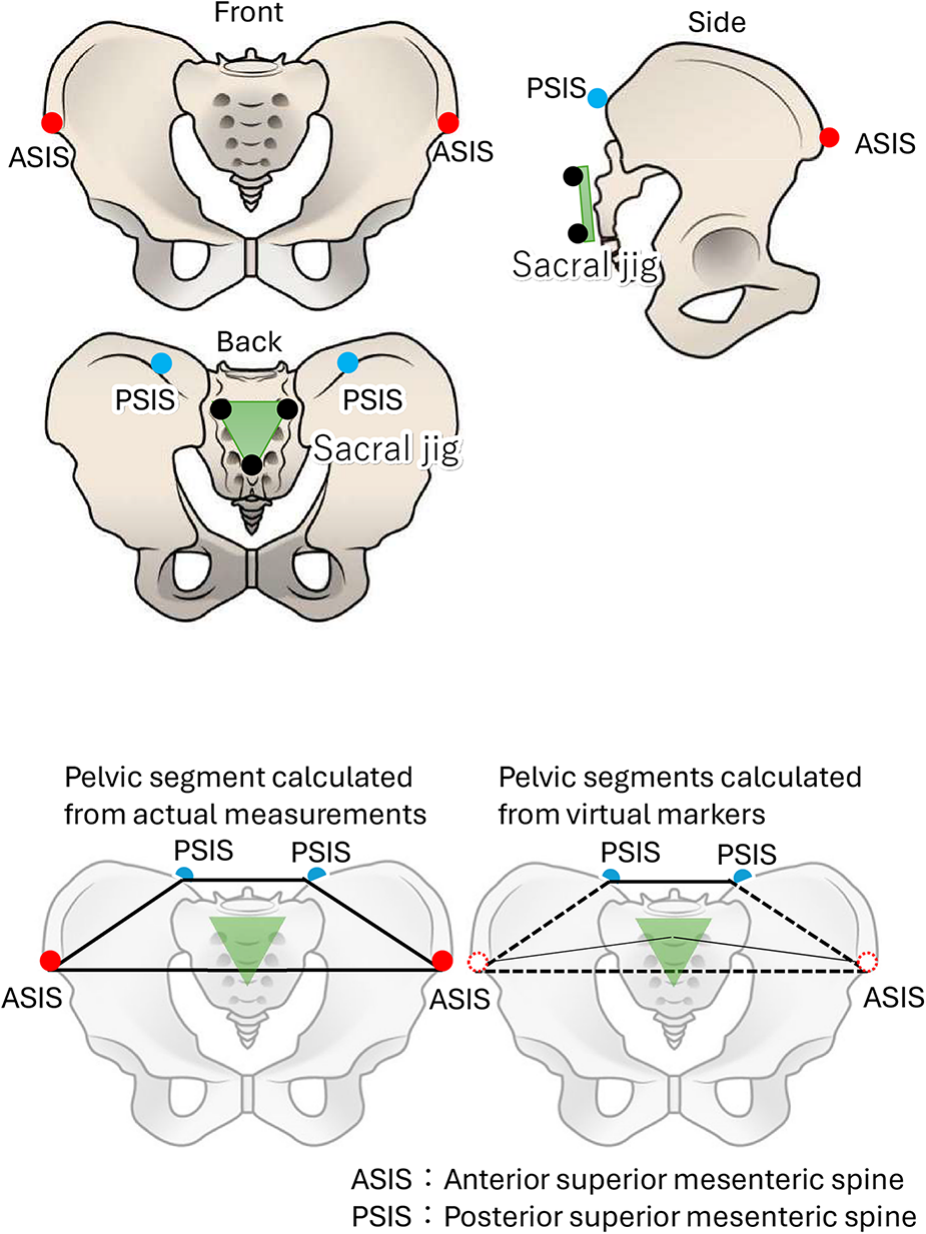
Pelvic marker affixing location (upper panel) and pelvic segment composition (bottom panel).

To define the pelvic segment, markers were positioned on the bilateral anterior superior iliac spines (ASIS) and the bilateral posterior superior iliac spines. However, during activities involving forward bending, the ASIS often became obscured by the body, rendering it invisible to the motion capture system. To address this issue, virtual markers were employed. The distance between the sacral jig and ASIS was accurately measured while the subject stood statically. Subsequently, when the ASIS markers became obscured during motion, we reconstructed the pelvic segments by employing virtual markers based on the previously recorded measurements (see Figure 4).

Marker position coordinate data, captured by a three-dimensional motion analyzer, were labeled and interpolated using the VICON NEXUS system. Based on guidelines from prior studies, the cutoff frequencies were established at 6 Hz for marker positional coordinate data and 18 Hz for ground reaction forces data. Subsequently, a Butterworth Lowpass filter was applied to smooth the data (17). Following this preprocessing, various kinematic and kinetic indices were extracted for analysis.

### 2.2 Participants

Seventeen physical therapy students (9 females, 8 males) and seven faculty members (all males) from the university were recruited as novice and expert participants, respectively, for the experiment. Prior to commencement, an overview of the experiment was provided, and only those who consented were included. There were no missing data, and all participants’ data were utilized. The novice participants, ranging from first to fourth-year students, had an average age of 20.7 ± 0.9 years. They had been introduced to support techniques for standing movements through lectures and practical classes. While all students had received instruction on these support techniques, none had yet taken the national physical therapist exam or obtained a physical therapist license. Furthermore, students from the first to third years lacked any clinical experience in physical therapy, and only fourth-year students had briefly encountered clinical situations during their practicum. The expert participants, all faculty members teaching physical therapy, were nationally licensed and possessed over 15 years of clinical experience, with an average age of 46.7 ± 13.7 years. They instructed students in physical therapy practices daily and provide support at hospitals or facilities at least once a month.

Third- and fourth-year physical therapy students (5 females, 12 males) were recruited to serve as collaborators in the experiment. These collaborators, with an average age of 21.0 ± 1.4 years, were instructed to simulate the role of patients throughout the experimental procedures. In this study, students were recruited as collaborators instead of actual patients in order to control for fatigue factors associated with performing five repetitions of sitting and standing.

### 2.3 Procedure

The experiment was conducted in pairs, consisting of either one expert participant paired with a collaborator, or one novice participant paired with a collaborator. Both expert and novice participants were equipped with the wearable eye tracker. Calibration of this device involved the experimenter presenting a card featuring a calibration point positioned 50–100 cm in front of the participants’ eyes. This process was essential for aligning and correcting the eye gaze data and the camera image.

**Figure 5:**
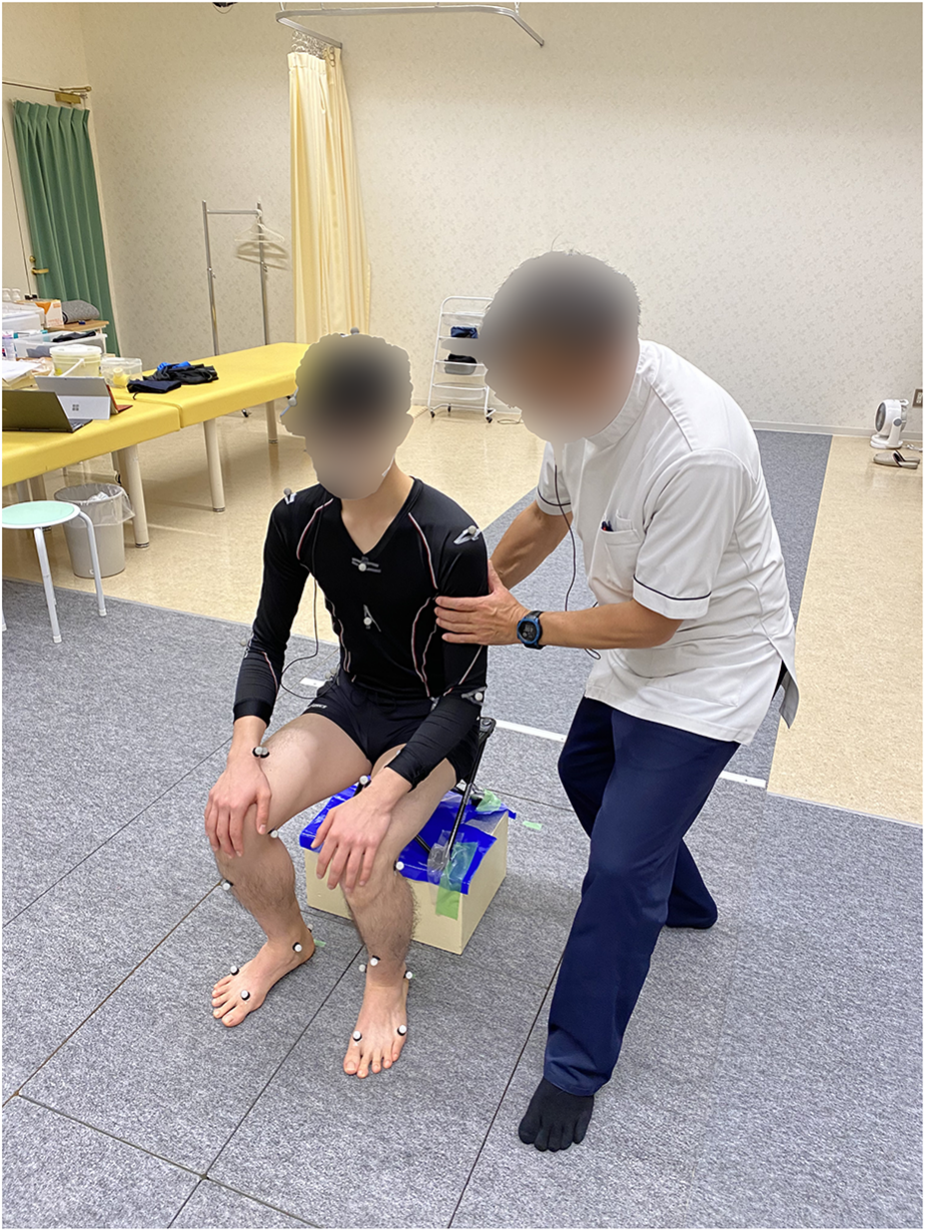
The standing positions during the experiment.

The collaborator was positioned on a chair situated directly above ground reaction force plates, with instructions to place their feet centrally on each plate. Expert and novice participants were positioned to the left of the collaborator, ensuring they stood outside the boundary of the ground reaction force plates (see Figure 5). This arrangement was designed to isolate the measurement of the collaborator's COM changes during standing and sitting activities.

Once in position, both expert and novice participants were directed to assist the collaborator in standing up from and sitting down in the chair, mimicking a clinical interaction by verbally cueing and physically supporting the collaborator. This support process involved a sequence of five repeated motions, where the collaborator was asked to stand up and then sit down five times consecutively. Data concerning eye gaze and motor indicators were collected throughout these repeated stand and sit cycles. Throughout the experiment, collaborators were instructed to respond naturally to the support provided by both expert and novice participants.

### 2.4 Measures

Gaze point data were collected using wearable eye trackers (Tobii Pro Glasses 3, Tobii). The analysis was conducted with the aid of specialized gaze analysis software (Tobii Pro Lab, Tobii). Coordinates within the first-person video were derived from the estimated gaze points recorded during the motions of assisting the collaborator to stand up.

The Areas of Interest (AOI) were defined as the head, neck, upper trunk, hip joint, knee joint, and foot. The AOI of head was defined as the range above the chin. The AOI of the neck was defined as the range from the chin to the acromion (up to the shoulder marker). The AOI of the upper trunk was set as the range of the upper 50% from the acromion to the hips. The AOI of the hip joint was defined as the line connecting the superior anterior iliac spine and superior posterior iliac spine (anterior and posterior hip trouser markers) and the proximal portion of the thigh long axis (up to halfway up the thigh). The AOI of the knee joint was defined as the distal portion of the femoral long axis (to the distal half of the thigh) and the proximal portion of the lower femoral long axis (to the proximal half of the lower legs) from the center of the knee joint. The AOI of the foot was set distal to the external capsule.

From the gazing point data and AOI, we obtained the total duration of fixation time to AOI, the number of fixations to AOI, the mean fixation time to AOI per visit, and the latency to fixate to AOI. The period for eye tracker data collection was set from the onset of the collaborator's initial stand-up motion to the moment the participant released their hand after the fifth stand-up motion. Fixation was defined as when two viewpoints were within a predefined minimum distance from each other (using the Tobii Fixation filter, or the same fixation point if the velocity was below a specific threshold when using the Tobii I-VT filter). The total duration of fixation time to AOI was calculated by dividing the total time spent fixating at each AOI by the time from the start to the end of the supported rise movement and converting it into a percentage. The number of fixations to AOI was defined as the number of fixations to each AOI. The mean fixation time to AOI per visit was calculated as the average duration of fixations per AOI visit, where a visit was defined as the period between the initial fixation on an AOI and the subsequent fixation outside that AOI. The latency to fixate to AOI was defined as the time taken from the start of the measurement to the first fixation on each AOI. Since the gaze data exhibited no significant noise and maintained high quality, all data were used without any deletions.

To analyze the measurement data, VICON Body Builder (ver. 3.6.4, VICON) was utilized. The human body was segmented into ten distinct parts: trunk, pelvis, left and right upper limbs, left and right thighs, left and right lower legs, and left and right feet. This segmentation was based on the coordinate data from the 37 infrared reflective markers previously described, forming a rigid body link model. However, during the standing motion, significant displacement of the markers at the superior anterior iliac spine, which were used to define the pelvis segment, was observed due to hip flexion movements. To address this, both superior anterior iliac spines were initially defined in a stationary standing position on a jig attached to the sacrum. These coordinate values were then used to calculate the virtual markers of the right and left ASIS within the spatial coordinate system during standing movements (Figure 4). The pelvic segment was thus modeled using three points: the upper anterior iliac spine and the right upper posterior iliac spine at these virtual markers. Three-dimensional (3D) joint angles were calculated using Euler angles. The orientation of each segment around the *x*-axis relative to the spatial coordinate system was defined as anterior-posterior tilt or flexion/extension. The positive direction for these movements was designated as anterior tilt, flexion, and dorsiflexion.

**Figure 6:**
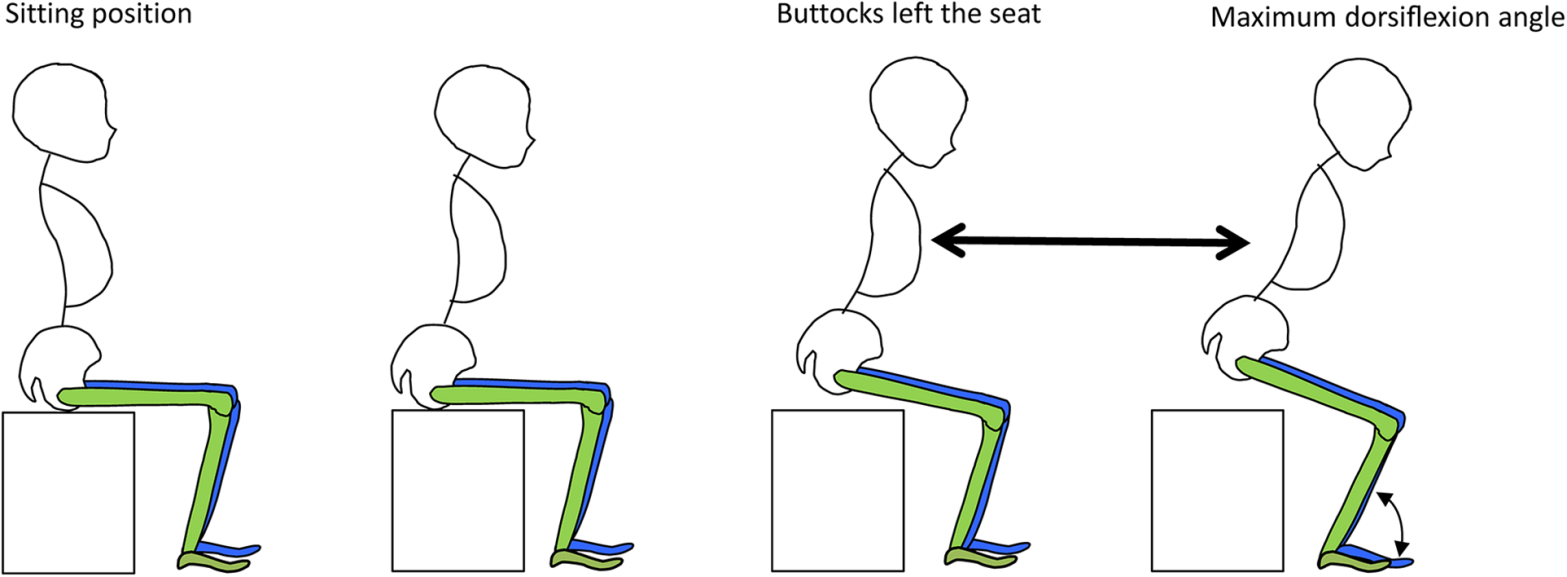
COM velocity. The distance of the forward movement of the center of gravity from the moment of detachment (the moment when the vertical component of the floor reaction force on the seat surface becomes zero) to the maximum dorsiflexion of the ankle joint was divided by the time required.

The moment the vertical directional force recorded by the two ground reaction force plates positioned behind the chair reached 0 N was defined as the point at which the hallux detached from the seat of the chair. The estimated the COM for each body segment was determined based on the positions of infrared markers attached to the body surface. Subsequently, the COM for the entire body was calculated using a weighted averaging method, which took into account the ratio of body segment weights (see Figure 6). Movement along the *y*-axis of the spatial coordinate system was categorized as forward or backward, with forward movement defined as positive direction movement.

**Figure 7:**
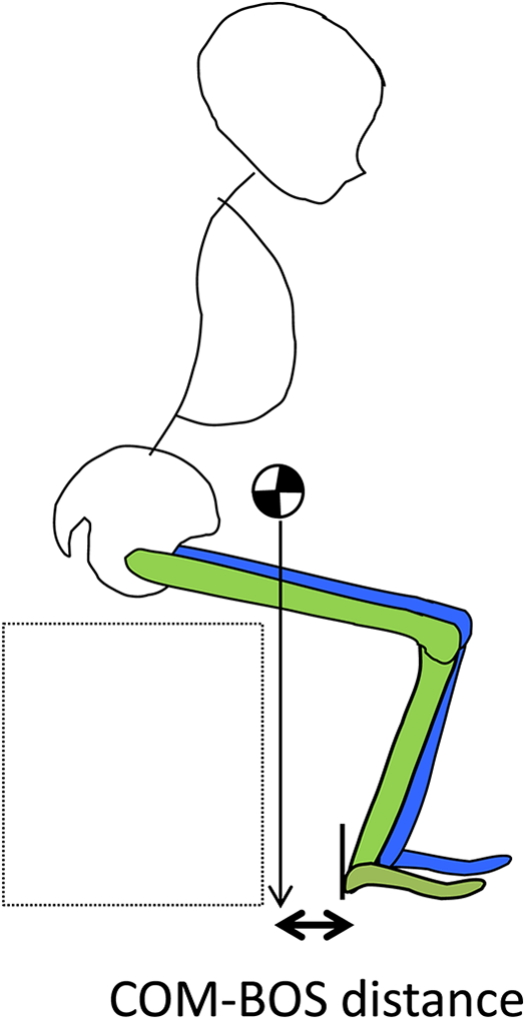
COM-BOS distance.

Next, the position of the infrared reflective marker on the heel was defined as the trailing edge of the BOS created by the foot, and the distance between the COM and the trailing edge of the BOS at the moment of leaving the foot was calculated (see Figure 7).

The fidelity of the procedure of the experimenter was evaluated using the following checklist: (a) the participant verbally called out to the collaborator while providing support, (b) the participant assisted the collaborator to stand up and sit down a total of five times, and (c) both the participant and the collaborator maintained their assigned positions throughout the duration of the experiment. Fidelity reached 100%.

## 3 Results

**Figure 8:**
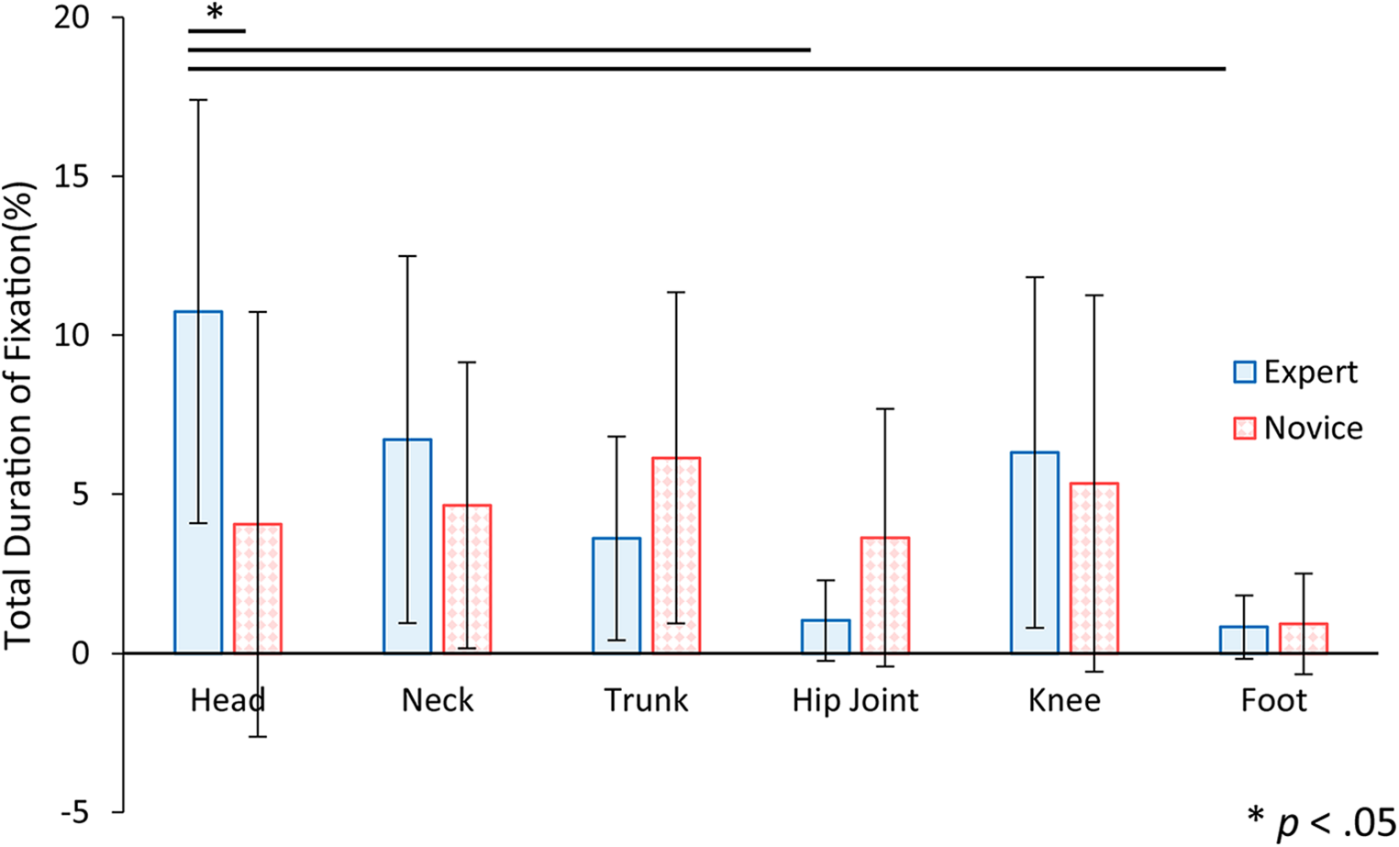
The total duration of fixation time to AOI for expert and novice participants. Error bars indicate standard deviation. Statistical analysis showed that there was a significant difference between experts and novices in the head region. There were also significant differences in the expert factor between head and hip joint, and between head and foot.

Table 1 shows the mean and standard deviation of the total duration of fixation time to AOI, the number of fixations to AOI, the mean fixation time to AOI per visit, and the latency to fixate to AOI for the expert and novice participants. Figure 8 shows the mean and standard deviation of the total duration of fixation time to AOI, comparing the expert and novice participants. To determine whether the total duration of fixation time to AOI varied based on the observer's experience and the specific body part observed, a two-factor mixed-design analysis of variance was conducted. The dependent variable was the total fixation time, with the experience of the observer and the AOI as factors. The results indicated significant differences for the main effect and the interaction of the AOI factor [*F* (5, 110) = 4.90, *p* < .01; *F* (5, 110) = 2.48, *p* < .05], suggesting variations in fixation durations across different body parts. However, no significant difference was observed for the main effect of the experience factor [*F* (1, 22) = 0.53, *n.s.*]. Further analysis through simple main effect tests showed that experts spent significantly more time gazing at the head region compared to novices. Additionally, when considering the AOI for the expert group alone, multiple comparisons using the Bonferroni method revealed that the duration of fixation on the head region was significantly longer than on the hip and foot regions. These findings suggest that experts prioritize the head region during observation, possibly due to its critical role in providing important cues for assessment, which they may actively utilize more than information from the lower body regions.

**Table 1:** Means and standard deviations of indicators related to eye gaze.

	Expert (*n* = 7)	Novice (*n* = 17)	
Total duration of fixation time to AOI			
Head	10.75 (6.66)	4.06 (6.68)	*
Neck	6.72 (5.77)	4.65 (4.50)	
Trunk	3.61 (3.20)	6.15 (5.21)	
Hip joint	1.03 (1.27)	3.63 (4.05)	
Knee	6.32 (5.52)	5.34 (5.92)	
Foot	0.82 (1.00)	0.93 (1.58)	
Total	29.25 (8.98)	24.76 (14.56)	
The number of fixations to AOI			
Head	25.71 (13.27)	8.65 (13.73)	*
Neck	17.29 (13.20)	12.18 (14.74)	
Trunk	7.43 (6.37)	12.18 (9.71)	
Hip joint	2.57 (3.02)	6.65 (17.73)	
Knee	14.57 (10.62)	9.12 (11.13)	
Foot	2.86 (3.36)	2.00 (2.99)	
Total	11.74 (2.77)	8.46 (6.79)	
The mean fixation time to AOI per visit			
Head	166.00 (48.53)	108.00 (92.93)	
Neck	135.57 (48.01)	108.00 (66.35)	
Trunk	178.71 (93.85)	129.12 (49.42)	
Hip joint	97.43 (92.98)	130.29 (108.75)	
Knee	161.57 (114.07)	198.59 (243.09)	
Foot	56.29 (67.30)	59.88 (91.09)	
Total	132.60 (53.26)	122.31 (67.43)	
The latency to fixate to AOI			
Head	10,510.57 (8,097.94)	9,140.35 (8,527.18)	
Neck	9,557.86 (3,838.62)	11,136.35 (8,948.31)	
Trunk	16,244.29 (10,688.89)	15,683.76 (11,581.14)	
Hip joint	8,470.57 (11,212.48)	9,088.71 (10,543.26)	
Knee	17,375.14 (20,565.57)	11,517.71 (10,591.44)	
Foot	2,354.00 (3,549.93)	11,374.53 (19,617.48)	

Mean (Standard deviation).

* *p* < .05.

**Figure 9:**
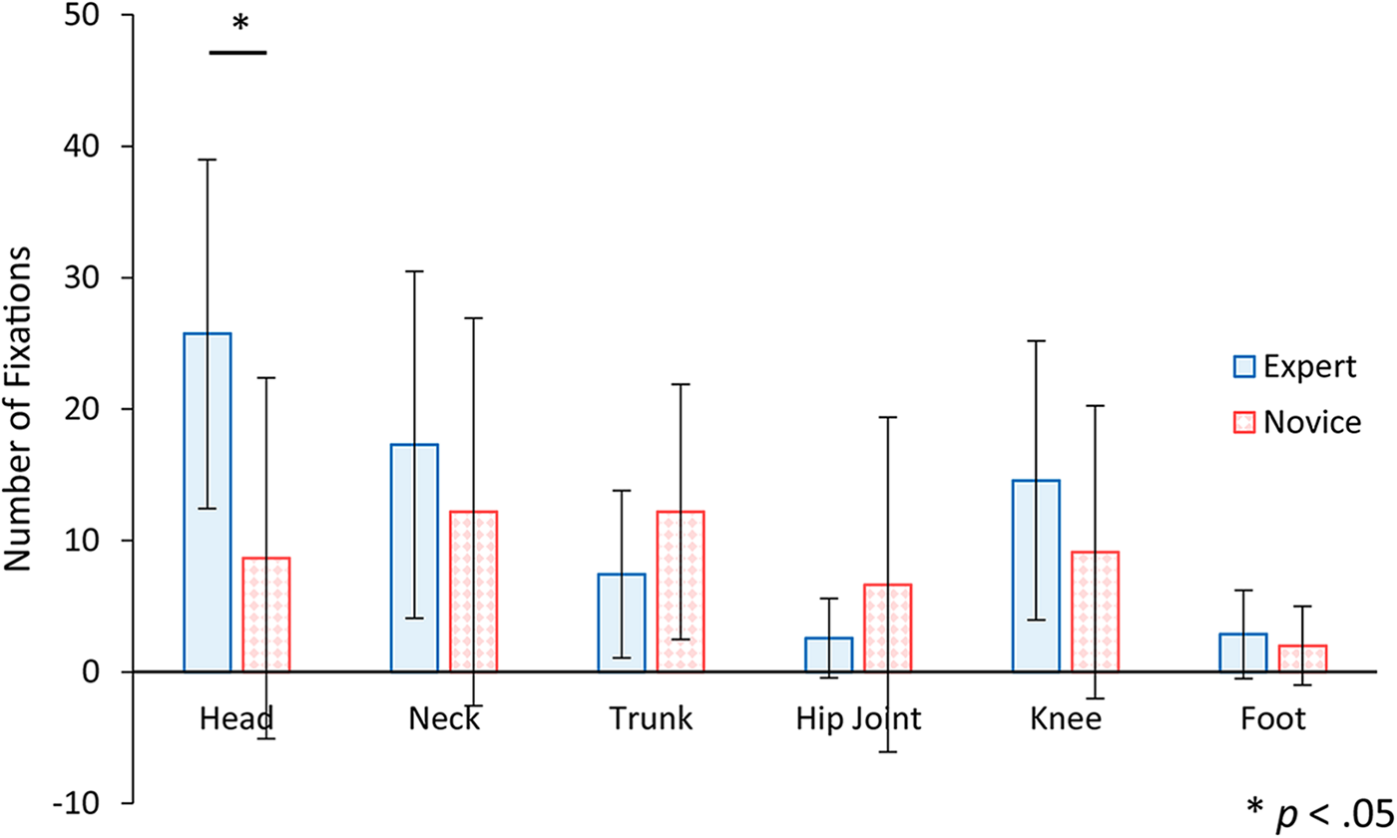
The number of fixations to AOI for expert and novice participants. Error bars indicate standard deviation. Statistical analysis showed that there was a significant difference between experts and novices in the head region.

Figure 9 shows the mean and standard deviation of the number of fixations to AOI, comparing the expert and novice participants. To assess whether the number of fixations to AOI varied according to the observer's experience level and specific body parts, a two-factor mixed-design analysis of variance was employed. The analysis considered the experience of the observer and the AOI as factors. The results indicated significant differences for the AOI factor and its interaction [*F* (5, 110) = 5.87, *p* < .01; *F* (5, 110) = 2.89, *p* < .05], pointing to variations in fixation counts across different body parts. Conversely, no significant difference emerged for the main effect of the experience factor [*F* (1, 22) = 1.40, *n.s.*]. Further analysis through simple main effect tests showed that experts exhibited a significantly higher number of fixations on the head area compared to novices. Additional multiple comparisons using the Bonferroni method within the expert group indicated that the number of fixations on the head region was significantly higher than those on the hip and foot regions. These findings suggest that experts do not merely fixate on the head once for a prolonged period but consistently re-check the head's position during the support process, emphasizing its importance in their assessment and support strategies.

**Figure 10:**
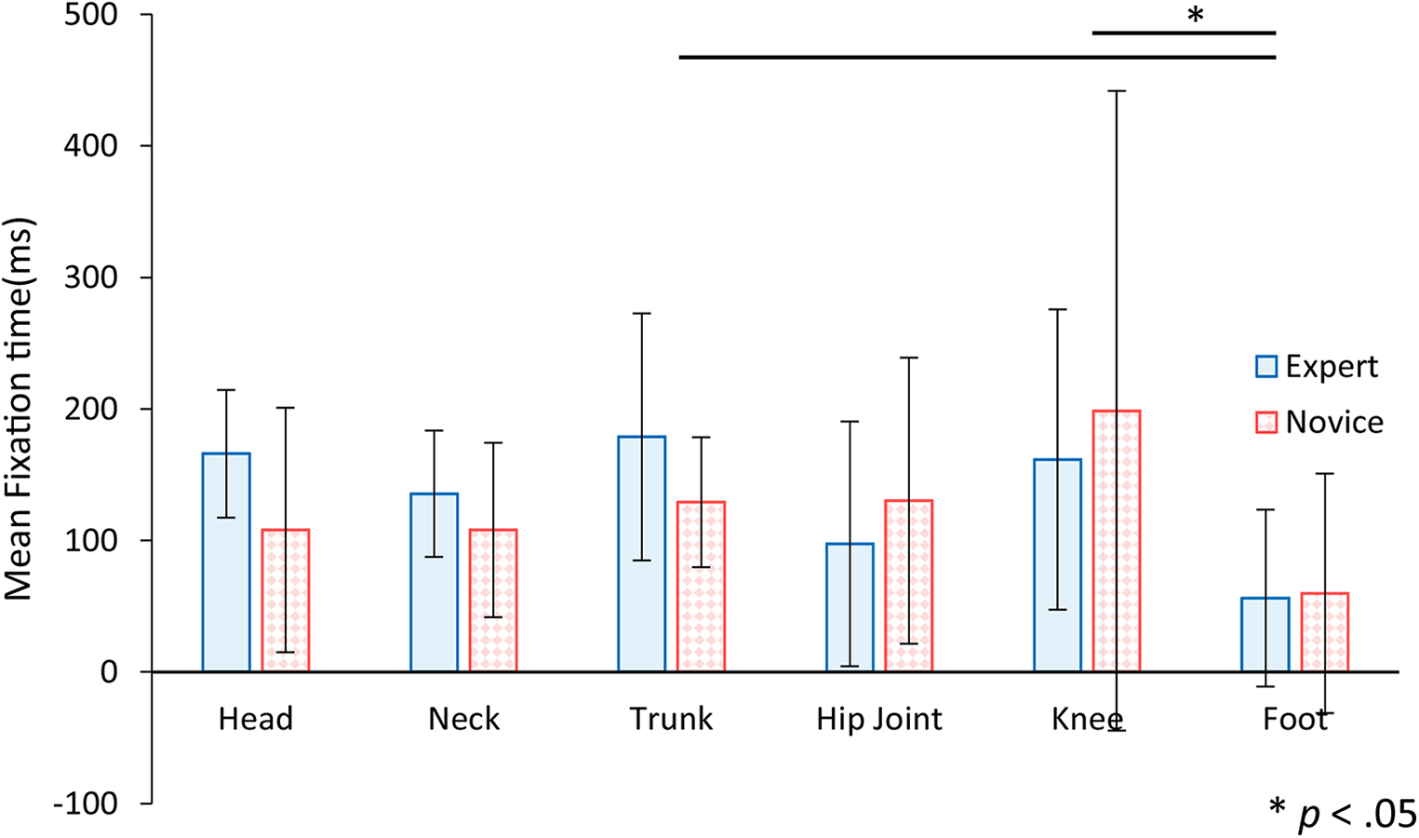
The mean fixation time to AOI per visit for expert and novice participants. Error bars indicate standard deviation. Statistical analysis showed significant differences between trunk and foot, and between knee and foot.

Figure 10 shows the mean and standard deviation of the mean fixation time to AOI per visit, comparing the expert and novice participants. An analysis of variance for a two-factor mixed design, incorporating factors of experience and AOI, was performed on the mean fixation time to AOI per visit. The results indicated a significant main effect for the AOI factor [*F* (5, 110) = 2.87, *p* < .05], showing variations in fixation duration across different AOIs. However, no significant differences were observed for the main effect or the interaction of the experience factor [*F* (1, 22) = 0.12, *n.s.*; *F* (5, 110) = 0.70, *n.s.*]. Further analysis through multiple comparisons using the Bonferroni method demonstrated that the mean fixation time per visit to the foot was significantly shorter compared to the upper trunk and knee. This pattern of results, with no significant differences found in the head region, is consistent with the observed frequent but brief checks of the head's position during assistance, suggesting an efficient monitoring strategy by the participants.

**Figure 11:**
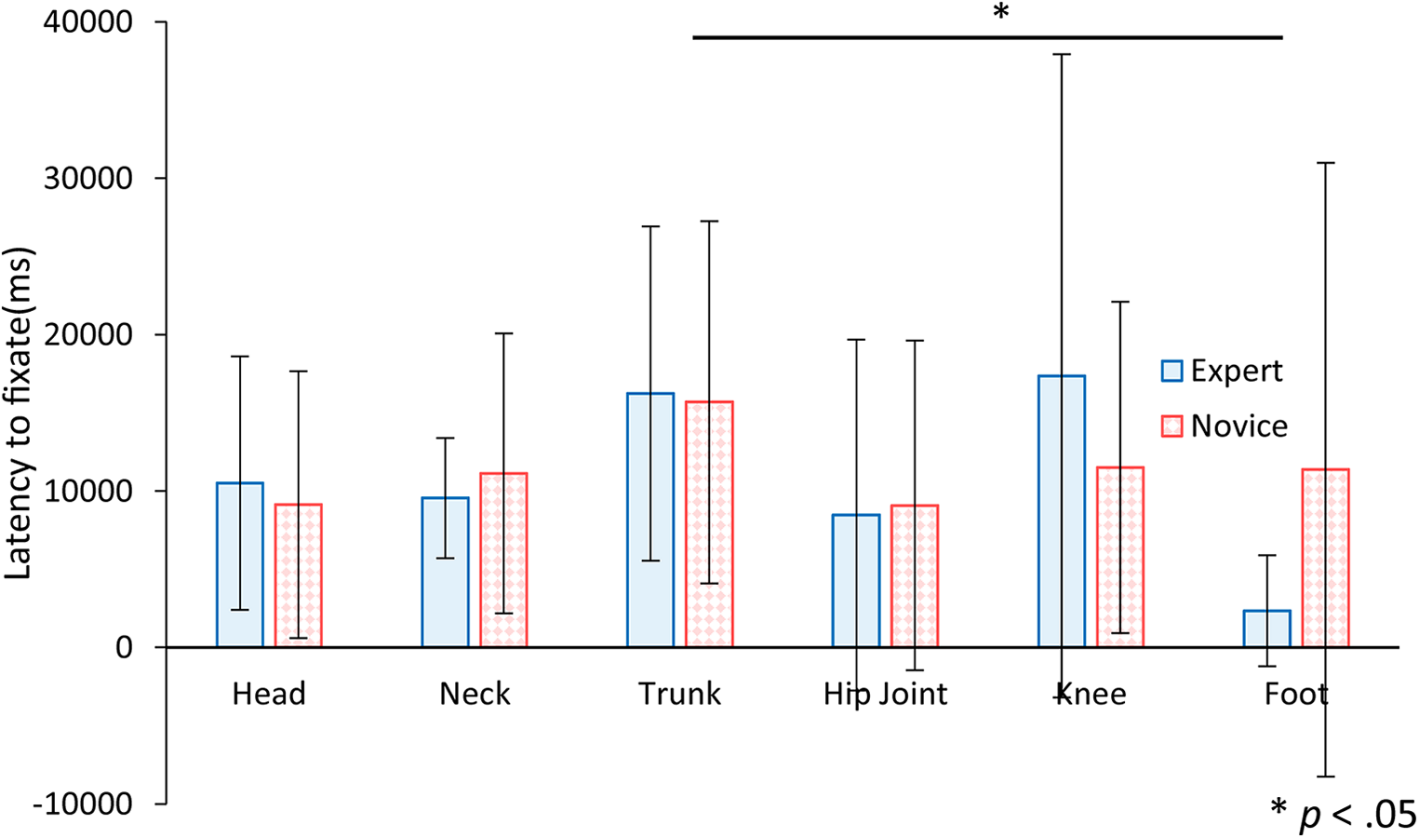
The latency to fixate to AOI for expert and novice participants. Error bars indicate standard deviation. Statistical analysis showed a significant difference between trunk and foot.

Figure 11 shows the mean and standard deviation of the latency to fixate to AOI, comparing the expert and novice participants. An analysis of variance for a two-factor mixed design, incorporating experience and AOI as factors, was conducted to assess the latency to first fixate on an AOI. The results revealed a significant main effect for the AOI factor [*F* (5, 110) = 2.52, *p* < .05], indicating variability in the time taken to first fixate on different AOIs. However, there were no significant differences in the main effect or interaction for the experience factor [*F* (1, 22) = 0.02, *n.s.*; *F* (5, 110) = 1.24, *n.s.*]. Further analysis using multiple comparisons with the Bonferroni method showed that the initial fixation on the foot occurred significantly earlier than on the upper trunk. These findings suggest a tendency among participants to check the position of the feet first upon starting the experiment, although no consistent pattern was observed in fixation times across all participants.

**Figure 12:**
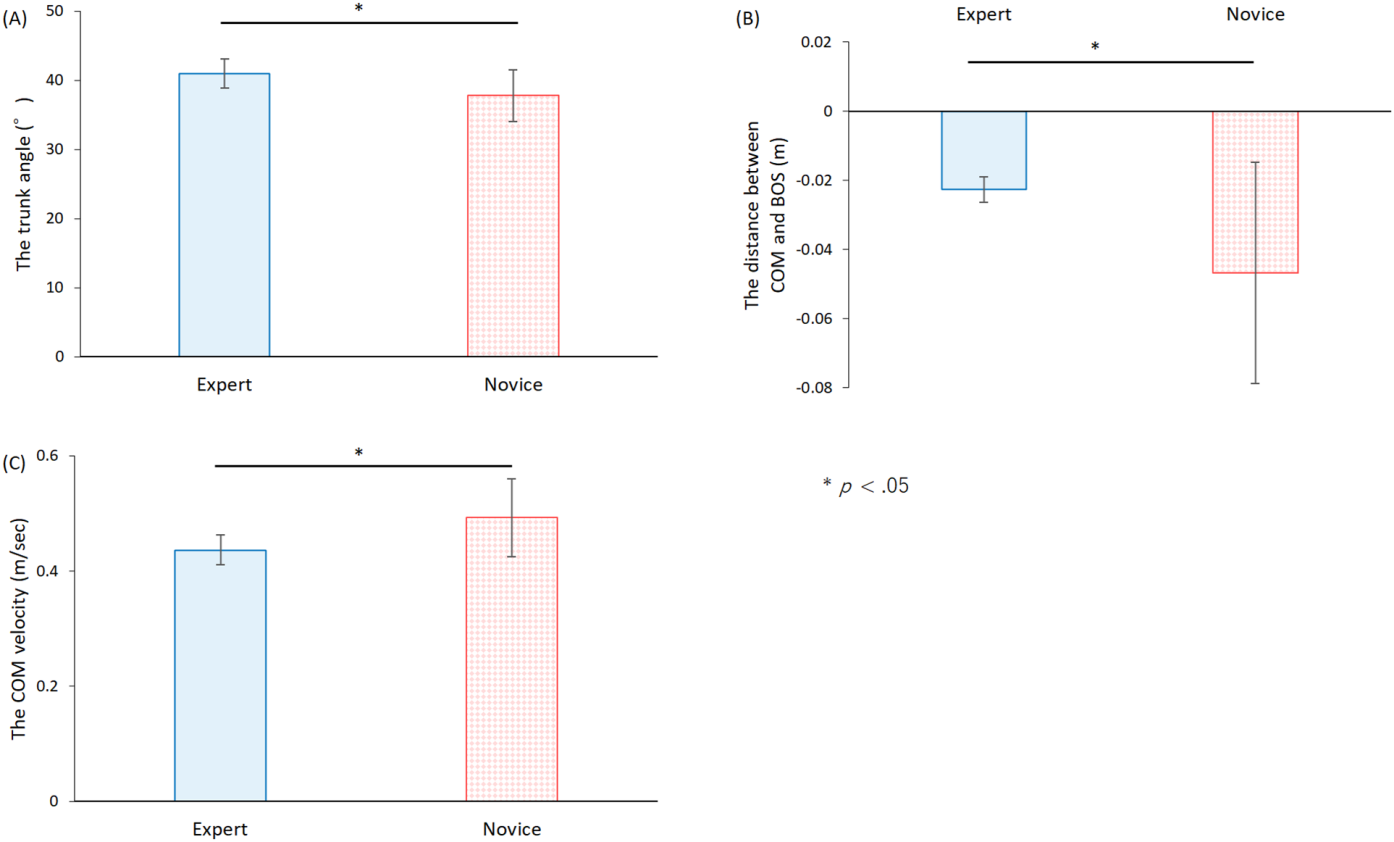
Kinematic indices of experimental collaborators receiving standing and sitting support. Error bars indicate standard deviation. COM; Center of mass. BOS; Base of support. **(A)** The deepest angle of the trunk forward during each trial. **(B)** The distance between the COM and the trailing edge of the BOS. **(C)** The COM movement velocity in the front-back direction during the rising motion.

Table 2 presents a comparison between experts and novices regarding the deepest forward-leaning trunk angle, the distance between the COM and the BOS, and the velocity of the COM. Figure 12A displays the mean and standard deviation of the deepest forward-leaning trunk angle across the five trials. The analysis revealed that the deepest forward-leaning trunk angle by the expert was significantly greater than that by the novice [*t* (118) = 5.88, *p* < .01]. This suggests that experts supported the collaborator to adopt a posture with their trunks more deeply bent during the stand and sit movement.

**Table 2:** Basic statics of kinematic indices of experimental collaborators.

	Expert (*n* = 7)	Novice (*n* = 17)	
The truck angle (degree)			
Range	36.54–45.76	30.05–50.66	
Mean (SD)	41.0 (2.1)	37.8 (3.7)	**
Median (Q1–Q3)	41.06 (39.94–42.53)	38.01 (35.23–40.20)	
The distance between COM and BOS (m)			
Range	−0.0311 to −0.0178	−0.329 to −0.0199	
Mean (SD)	−0.023 (0.003)	−0.047 (0.032)	**
Median (Q1–Q3)	−0.0211 (−0.0247 to −0.0200)	−0.0447 (−0.0492 to −0.0413)	
The COM velocity (m/sec)			
Range	0.396–0.489	0.319–0.661	
Mean (SD)	0.436 (0.026)	0.492 (0.067)	**
Median(Q1–Q3)	0.431 (0.415–0.457)	0.502 (0.443–0.532)	

SD, standard deviation; Q, quartile.

** *p* < .01.

Figure 12B illustrates the mean and standard deviation of the distance between the trailing edge of the base support surface and the position of the COM at the moment the buttocks left the seat surface during the trial. The analysis indicated that the distance between the trailing edge of the BOS and the position of the COM was significantly shorter for experts compared to novices [*t* (118) = 6.81, *p* < .01]. This finding suggests that experts brought the collaborator's bodies closer to the COM before initiating the rise, potentially enhancing stability and control during the movement.

Figure 12C displays the mean and standard deviation of the COM velocity in the front-back direction during the rising motion. Statistical analysis revealed that the COM velocity in the front-back direction during the rising motion was significantly slower for experts compared to novices [*t* (118) = 6.52, *p* < .01]. This result suggests that experts facilitated a more controlled and slower movement of the COM, providing support that allowed subjects to adjust their motion gradually.

## 4 Discussion

The aim of this study was to investigate differences in eye movement and assistance methods between expert and novice physical therapists during actual standing movements. Physical therapists adjust their interactions dynamically based on the visual information gathered from observing patients. Findings from this study revealed that expert participants gazed at the head area longer and more frequently than novice participants, suggesting a more focused observation of this region. Moreover, experts demonstrated a distinctive approach in the physical assistance they provided. Before initiating the stand, they significantly flexed their trunks to align the COM closely with the BOS. This preparatory action was followed by a gradual push to move the COM slowly, thereby minimizing the load on the collaborator. During the assistance, experts also induced a greater flexion in the collaborator's trunk angle compared to novices, influencing the collaborator's posture and movement during the rise. Most existing studies comparing the gaze behaviors of experts and novices have utilized static, prerecorded videos or images, which may not adequately represent the dynamic decision-making processes in therapeutic settings. Unlike these studies, our research highlights that the support strategies employed by experts differ from those of novices, indicating that using uniform visual stimuli may not effectively capture the nuanced expertise of professional gaze in action. This study contributes to the field by integrating first-person gaze measurement with motion analysis, an approach previously underexplored in rehabilitation research.

We hypothesize that experts focus extensively on the head to accurately determine the position of the COM along the sagittal plane and to precisely time the upward push at the hips. When assisting a collaborator in standing and sitting, it is crucial for experts to initiate the upward push at the waist only after the collaborator's COM has moved sufficiently forward. This strategy allows the collaborator to stand with minimal assistance. Attempting to push the waist before the COM is adequately forward compels both the therapist and the collaborator to exert more force, thereby increasing the burden significantly. In this study, the distance between the BOS and COM at the moment of disengaging support at the waist was greater when novices provided support compared to when experts did. Additionally, the angle of trunk forward tilt was less pronounced in novices’ attempts. These observations suggest that novices might initiate the waist push prematurely, when the collaborator has not leaned forward adequately, positioning the COM behind the collaborator. Consequently, a greater force was required to achieve standing, resulting in a faster forward COM velocity compared to that observed with experts. To mitigate such rapid shifts in COM, continuous monitoring of the COM line is essential during support, ensuring that the collaborator has moved forward sufficiently. Experts likely assess the position of the COM by observing the relative positions of the collaborator's head and feet. If the push is timed when the head is positioned more forward relative to the feet, the collaborator can stand more easily with less exertion. This observation may explain why experts spend more time focusing on the head, as it provides critical visual cues necessary for optimizing support timing and reducing the physical effort required.

Another interpretation of experts’ frequent focus on the facial region is that they may be evaluating the effectiveness of their assistance based on the patient's facial expressions. Many patients, particularly those with cognitive or communicative impairments, are unable to verbally articulate their discomfort. For instance, patients with dementia often struggle to express physical pain and discomfort verbally (18, 19). Similarly, approximately one-third of stroke patients suffer from aphasia accompanied by motor paralysis, significantly limiting their communicative abilities (20). Furthermore, nearly half of individuals with traumatic brain injury exhibit cognitive dysfunction that impedes their communication (21). In such cases, physical therapists may rely on facial expressions as critical indicators of pain and discomfort (19). It is speculated that experts, drawing on their extensive clinical experience, devote more attention to patients’ facial expressions to gauge whether their support techniques are effective and well-tolerated. This observation underscores the importance of non-verbal cues in the therapeutic setting, especially when verbal communication is compromised.

In this study, we utilized a wearable eye tracker to directly measure the gaze patterns of physical therapists as they assisted patients, providing a unique perspective on real-time therapeutic interactions. This contrasts with the approach taken by Hayashi et al. (14), who analyzed eye movements of participants watching prerecorded patient videos from a third-person perspective. Our study reports findings that align with some of the dependent variables in Hayashi et al. (14) while showing different results for others. Both studies demonstrated that experts had more frequent gazes and tended to frequently fixate on the AOI. However, while Hayashi et al. (14) reported shorter gaze durations in AOI, such as paralyzed limbs, our study showed that experts spent longer periods gazing at AOI, such as the head. In our study, gaze data were collected from the therapists’ own first-person perspective while they supported the collaborator, whereas Hayashi et al. (14) collected data from participants observing a third-person video, which may not fully capture the dynamic decision-making processes involved in live therapeutic scenarios. In the fixed third-person perspective videos, even if participants wanted to spend more time observing a specific area, the desired body part might be obscured by the collaborator's posture, and they would not be able to zoom in for a closer look. In contrast, a free first-person view allows participants to reposition themselves or approach the patient for a closer look, even if the patient moves. This difference in the characteristics of video footage and eye-tracking methods may explain why experts in our study demonstrated longer total duration of fixation time. However, it is important to acknowledge that the tasks themselves differ significantly between these studies. In Hayashi et al. (14), participants were asked to observe patient videos and describe symptoms, which involves different cognitive and observational processes than those involved in directly assisting a patient's movement. The quality and focus of the gaze observed in their study may have been influenced by the analytical nature of describing symptoms, which is quite different from the dynamic real-time adjustments required when physically assisting patient movement. When identifying symptoms, it may be necessary to quickly observe multiple body areas and selectively gather information rather than spending extended periods observing a single area. On the other hand, when providing actual supports, experts may need to gaze at relevant areas for longer periods to receive feedback on the outcomes of their support, leading to longer fixations on task-related areas.

The results suggest that gaze and movement analysis hold promise for improving therapist training programs and clinical practice. Future research could develop training focused on improving these specific assistance skills by identifying cues that distinguish experts from novices. For example, prior to hands-on training for novices in the standing up movement assistance practice, educators may have shown novices that when assisted by the experts, compared to novices, (1) Their gazing time is longer in the head region than in the lumbar or foot region, (2) The angle of forward tilt of the trunk during standing up was greater, (3) The educator can teach that the subject begins to stand up after moving her/his body slowly until she/he approaches the position of the COM. Based on this information, the educator can then demonstrate the assistance method to the novice as a model. Furthermore, in practical training for assisting the rising motion, a system can be developed that measures the gaze of the novice and provides feedback when the patient is pushed to stand at a time when the gaze is too little or too early to the head area, thereby gaining insight into gaze patterns and body movements during the interaction with the patient. This feedback can be obtained. This feedback could help bridge the performance gap between novices and experts, potentially leading to improved patient outcomes.

This study has three limitations. First, in this study, we recruited students rather than actual patients as collaborators. Their behaviors may not fully replicate those of actual patients, potentially leading to differences in how the participants would act when supporting real patients. For example, unlike actual patients, the students may spontaneously shift their COM forward or flex independently. When such movements occur naturally, it is generally appropriate for physical therapists to observe rather than forcefully support the collaborator's movement. In contrast, with actual patients who are not expected to initiate standing movements independently, the experts need to provide active support, and the expert's gaze patterns are also expected to differ accordingly. Therefore, it remains uncertain whether the methods of supporting participants to stand or the gaze patterns observed in this study are identical to those in real clinical settings. Second, the collaborator could see whether it was an expert or novice participant who was supporting him or her. In this study, both experts and novices participated in the experiment simultaneously. However, to prevent the experimental effects, it is necessary to conduct the sessions independently in separate rooms with sufficient time intervals between them. Finally, we were unable to examine peripheral vision. In the present study, the total fixation time of experts was shorter than that of novices. This indicates that experts may acquire more information in a shorter time, and also that they may obtain information by peripheral vision rather than by staring at a single point. Due to the specifications of the experimental equipment, we were only able to examine the characteristics of central vision.

## Data Availability

The raw data supporting the conclusions of this article will be made available by the authors, without undue reservation.
